# Binturong (*Arctictis binturong*) and Kinkajou (*Potos flavus*) Digestive Strategy: Implications for Interpreting Frugivory in Carnivora and Primates

**DOI:** 10.1371/journal.pone.0105415

**Published:** 2014-08-26

**Authors:** Joanna E. Lambert, Vivek Fellner, Erin McKenney, Adam Hartstone-Rose

**Affiliations:** 1 Department of Anthropology, The University of Texas at San Antonio, San Antonio, Texas, United States of America; 2 Department of Animal Science, North Carolina State University, Raleigh, North Carolina, United States of America; 3 Department of Biology, Duke University, Durham, North Carolina, United States of America; 4 Department of Cell Biology and Anatomy, University of South Carolina, Columbia, South Carolina, United States of America; Duke University School of Medicine, United States of America

## Abstract

Exclusive frugivory is rare. As a food resource, fruit is temporally and spatially patchy, low in protein, and variable in terms of energy yield from different carbohydrate types. Here, we evaluate the digestive physiology of two frugivorous Carnivora species (*Potos flavus, Arctictis binturong*) that converge with primates in a diversity of ecological and anatomical traits related to fruit consumption. We conducted feeding trials to determine mean digestive retention times (MRT) on captive animals at the Carnivore Preservation Trust (now Carolina Tiger Rescue), Pittsboro, NC. Fecal samples were collected on study subjects for *in vitro* analysis to determine methane, pH, and short chain fatty acid profiles; fiber was assayed using standard neutral detergent (NDF) and acid detergent (ADF) fiber methods. Results indicate that both carnivoran species have rapid digestive passage for mammals that consume a predominantly plant-based diet: *A. binturong* MRT = 6.5 hrs (0.3); *P. flavus* MRT = 2.5 hrs (1.6). *In vitro* experiments revealed no fermentation of structural polysaccharides – methane levels did not shift from 0 h to either 24 or 48 hours and no short chain fatty acids were detected. In both species, however, pH declined from one incubation period to another suggesting acidification and bacterial activity of microbes using soluble carbohydrates. A comparison with primates indicates that the study species are most similar in digestive retention times to *Ateles* – the most frugivorous anthropoid primate taxon.

## Introduction

Most specialist mammalian frugivores are found in the tropics where fruit is more likely to be available year-round compared to regions with more extreme seasons. Exclusive frugivory is rare even in the tropics, however, as fruit is a temporally and spatially patchy resource, generally low in protein (<1% N or <6% crude protein dry mass), and variable in terms of energy yield from carbohydrates [1]–[4]. Thus, while often described as a “high-quality” food [5]–[7], fruit is not without its limitations and frugivorous mammals must have adaptations for balancing macronutrient intake, modulating physiology to offset deficiencies, or switching foods altogether to cope with limiting availability and nutrient density [8]–[17]. While some adaptations for offsetting deficiencies or switching foods can be constrained by a species' phylogeny and anatomical baupläne [18], others, particularly digestive, are more plastic [19], [20]. Among arboreal (non-volant) placental mammals, only a few carnivoran (e.g., procyonid, viverrid) and some primate species (e.g., *Ateles, Pan*) come close to exhibiting almost exclusive frugivory [1], [13], [21]–[24].

Recent macronutrient analyses have demonstrated that highly frugivorous primate species (e.g., *Ateles* spp) target ideal protein to energy ratios by maintaining a fairly constant protein intake (as measured by dietary nitrogen; N) while allowing non-protein energy (carbohydrates + lipids) to vary as a function of nutritional composition of available foods [13], [14]. Other highly frugivorous mammal species (e.g., didelphid marsupials, pteropodid bats) maintain nitrogen balance by increasing total fruit intake (thereby increasing total dietary N intake) and decreasing retention times [3], [23]. However, increasing intake is done at the expense of digestive efficiency – i.e., extraction and uptake of macronutrients, especially carbohydrates. Moreover, rapid through-put can result in a “washing out” phenomenon in which endogenous (non-diet) protein sources such as gut epithelial cells, enzyme products, and bacterial cells are defecated and lost [23], [25], [26]. Hence, if nitrogen is limited in availability, rapid digestive passage is predicted as it facilitates higher intake of low N foods. However, the type of carbohydrates consumed by an animal – and how efficiently energy is extracted and utilized – will limit just how fast digestive passage can be.

Consumers utilizing monosaccharides (such as highly frugivorous birds) tend to have high intake and rapid digestive passage [8], [20], [27]. The longest digestive retention times in mammals are found in herbivores that rely on either fore- or hindgut microbial fermentation of polysaccharides, but even catalytic digesters that consume less refractory, shorter-chain carbohydrate molecules (e.g., oligo- or disacarrhides) require sufficient time for enzyme production and nutrient transporter uptake. Indeed, small intestinal rates of hydrolysis and absorption can be rate limiting [20]. Thus, there are trade-offs with regard to intake and digestive efficiency such that a mammal's digestive strategy represents a behavioral, physiological, and morphological solution to a set of constraints related to the nutritional content and digestibility of its diet [1], [28]–[30].

We evaluate rate-efficiency trade offs and digestive strategies in two frugivorous Carnivora: kinkajous (*Potos flavus*; Procyonidae) and binturongs (*Arctictis binturong*; Viverridae). We chose these taxa because of their potential for shedding light on the constraints of frugivory and digestive trade-offs in the order Primates – the other major group of arboreal (and non-volant) placental mammals in which high frugivory is found. Obligate frugivory has evolved twice within another placental mammal group, the order Chiroptera, in both the sub-order Megachiroptera (Old World bats – ‘flying foxes’) and neotropical family Phyllostomadidae [3], [31]. However, we focus on *P. flavus* and *A. binturong* because of their overall phenotypic and ecological convergence with primates and to avoid an additional confounding variable introduced by the constraints of flight. A broad comparative method focusing on species that have converged on similar strategies facilitates understanding of the role of phylogeny on animal adaptations [32]. Although nocturnal, *P. flavus* and *A. binturong* have converged with diurnal anthropoid primates on suites of features related to their tropical, arboreal frugivory, including – among others – the maintenance of a clavicle (for flexible arboreal locomotion), bunodonty, large incisors (relative to other carnivorans), broad rostra, orbital frontation, and prehensile tails [33]–[35]. Both study species live in rainforests with high primate biodiversity – *A. binturong* in South East Asia and *P. flavus* in the neotropics. The few long-term studies on *P. flavus* ecology indicate this species has a diet with the highest proportion of fruit of all non-volant placental mammals [22], [36]–[38]. No detailed physiological data on *P. flavus* digestion exist, although a lack of a caecum and fast digestive passage rates have been noted [19], [23], [36]. *Arctictis binturong* gastrointestinal anatomy has not been described, although they are believed to maintain the simple gut anatomy (small acid stomach, no ceacum or elaboration of colon) common to the Order Carnivora [29]. To our knowledge, there has been no standardized data collection on *A. binturong* diet, although anecdotal reports indicate very high levels of frugivory [21], [39]–[41].

Here, we present results on digestive kinetics, *in vitro* fermentation profiles (SCFA, pH, methane), and fiber digestibility in *P. flavus* and *A. binturong* fed standard diets. We evaluate results in light of previously collected data on primate digestive strategies in order to understand rate-efficiency trade offs and frugivory in a broader, comparative framework.

## Methods

### Ethics Statement

The research was reviewed and approved by the University of Texas at San Antonio Institutional Animal Care and Use Committee (IACUC protocol #MS005). The laboratory in which all fermentation experiments occurred was inspected and approved by the Biosafety Committee of Environmental Health and Safety Division (BC – EH&S) at North Carolina State University (NCSU). The NCSU BC – EH&S certified that the laboratory met their stated safety criteria and that all personnel involved were trained in appropriate microbiological techniques and laboratory safety procedures.

### Study subjects and their husbandry

We studied the digestive retention times (15 trials) and *in vitro* fermentation profiles (16 experiments) of two *P. flavus* and four *A. binturong* September 2008 – March 2010. All animals were housed at the non-profit institution Carnivore Preservation Trust (CPT) in Pittsboro, North Carolina (now known as Carolina Tiger Rescue). Only two *P. flavus* (one male, one female) were available for study (male: 17.9 yrs, 3.9 kg; female: 22 yrs, 4.6 kg). Of the 12 *A. binturong* housed at CPT, 4 (all male) were chosen for study based on health status and similarity in age (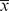 age: 13.75 yrs, range: 12.7–15.6 yrs) and weight (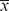 weight: 18.9 kg, range: 16.4–23 kg). The two *P. flavus* were housed individually in indoor enclosures measuring 3.6×2.1×3.1 m at an average ambient temperature of 23.3–26.6°C and under a 12L∶12D light regime. The four *A. binturong* were housed individually in large (5.5×3×4.5 m) outdoor enclosures with sleep boxes; because they were outside, day length and temperature varied across the four trial periods (e.g., 75 minute difference between September and March). All animals were fed standardized diets to which they were habituated for three days prior to each digestive retention and *in vitro* fermentation profile trial period (Table 1). The diet was based on regular zoo diet to avoid disruption of normal feeding routine.

**Table 1:** Macronutrient content of *Arctictis binturong* and *Potos flavus* diet, including extruded diet and the vegetative parts (leaves, petioles), reproductive parts (fruits, seeds), and modified roots (tubers) of domesticated plant species.

Food Category	Food Type	Gross Energy (kcal/g)	Crude Protein (%)	Crude Fat (%)	Crude Fiber (%)	NDF^3^ (%)	ADF^4^ (%)
Extruded forumulation^1^	Purina dog chow	3.1	22.6	5.23	11.1	23.3	14.5
Reproductive plant parts (fruit & seeds; raw, with peel) ^2^	Banana (*Musa sapientum*)	4.17	4.0	1.9	9.3	5.4	
	Cantaloupe (*Cucurmis melo*)	3.45	8.5	1.9	9.1		
	Kiwifruit *(Actinidia chinensis)*	4.12	5.8	2.6	17.0	16.2	12.6

Data from National Research Council (2003) and from guaranteed analyses from Purina. Nutritional content of extruded diet expressed as percent of ration; nutritional content of plant diet expressed as percent of dry matter (100% DM).

1 *Arctictis binturong*: 81 g/individual; *Potos flavus*: 81 g/individual.

2 *Arctictis binturong*: 228 g/individual; *Potos flavus*: 50 g/individual.

3 Neutral detergent fiber.

4 Acid detergent fiber.

### Digestive retention trials

The digestive retention trials involved feeding (at 1730 h) each study subject ten markers (each marker: 4×2×1 mm) concealed in a banana, following methods described by Lambert [42], [43] and replicated by several authors [44], [45]. Each individual was assigned its own marker color per trial, and the duration of time between marker ingestion and marker defecation was measured. Marker size was chosen based on the naturalistic observations of size of seeds swallowed by wild arboreal, frugivorous mammals in Africa and Asia [39], [46]. The markers were made of Pepperell Plastic Craft Cord - an inert, non-toxic plastic material known commonly as Gimp that has been approved by the Food and Drug Administration for use by young children.

We undertook four digestive passage trials on the male *P. flavus*, and four on the female. We conducted one digestive trial on two of the four male *A. binturong*; on male 3 we conducted two trials and on male 4 we conducted three trials.

After the animals consumed the marker-loaded bananas, the study subjects were fed their normal daily ration. We monitored all defecations until the markers were recovered, then screened all fecal material to determine whether the sample contained colored markers. Defecated markers were highly visible and readily identified and quantified. We recorded the time of defecation, the number and color(s) of markers per fecal sample, and assessed mean retention time of markers (MRT). MRT is the best estimate of digesta movement through mammalian gastrointestinal tracts [47], and is a measure of the average time of retention of all elements of the focal digesta (in this case, colored markers). MRT is calculated as the following in which m_i_  =  the number of markers excreted at the i^th^ defecation at time t_i_ after dosing: 
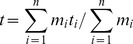



### Sample collection and laboratory analysis of *in vitro* bacterial fermentation profiles

We evaluated bacterial fermentation using standard *in vitro* methods on fecal samples collected from study subjects collected December 2008 – February 2010. A preliminary experiment (December 2008) in which only methane was analyzed indicated no fermentation activity; this initial run was terminated and the decision made to run three further experiments. Availability of staff and enclosure locations meant that we could not monitor all study subjects simultaneously. We were thus unable to collect sufficient fecal samples for *A. binturong* in the subsequent three experiments; we also only had sufficient fecal inoculum to undertake gas chromatography on culture samples at two time periods for each experiment (i.e., rather than three). After the preliminary experiment, we ultimately ran 3 *P. flavus* and 1 *A. binturong in vitro* experiments. The *P. flavus* analysis from the March 2009 collection was incubated for 0 and 48 h, and the September 2009 for 0 and 24 h. In the February 2010 binturong and *P. flavus* analysis, there was only enough fecal substrate to incubate for a 24 h period.

Following previously described methods [48], [49], we used controlled anaerobic methods to minimize exposure to air during fecal sample collection, transportation, manipulation, and maintenance. However, we cannot rule out that some aerobic exposure occurred, thereby lowering the reduction-oxidation potential of samples. Nonetheless, we have successfully employed these methods in previous experiments [49] and note, too, that anaerobic bacteria can have some ability to thrive after minimal oxygen exposure [50].

We used a sample of standardized diet of plant material and extruded food pellets to provide an appropriate growth medium in the fermentation tubes (Table 1). Samples were processed immediately upon arrival to the laboratory and within one hour of collection. Gas chromatography was undertaken for the by-products of bacterial fermentation of polysaccharides: short-chain fatty acids (SCFA) and methane; pH was also measured.

To monitor fermentation profiles, we used a batch system in which fecal inoculum was prepared to inoculate culture bottles [51], [52]. We replicated the methods we used previously for primates [49], with one exception: due to insufficient fecal sample quantity, the dilution of inoculum for *P. flavus* was 1∶6.5 instead of 1∶5. Following incubation, gas samples were withdrawn and analyzed for methane by gas chromatography. A pH measurement was taken following methane analysis, and 4-mL aliquots of unstirred fluid were sampled from each bottle and prepared for SCFA analysis.

Dry matter (DM) was determined following protocol outlined by the Association of Official Analytical Chemists (AOAC, method 945.15) [53]. Neutral detergent fiber (NDF) and acid detergent fiber (ADF; determined sequentially to NDF) were calculated according to the method of Van Soest and colleagues [54] using the Ankom 200 fiber apparatus (ANKOM Technology Corporation, Fairport, NY). Disappearance of DM, NDF and ADF was calculated using data from culture bottles at 0 and 24 or 48 h incubation periods.

## Results

### Digestive Passage Trials

The subjects readily consumed the marker-dosed bananas. All study subjects reached for the bananas manually and then immediately ingested them. The total recovery rate of markers swallowed by *P. flavus* was 97.5% (78/80), and by *A. binturong* was 91.4% (64/70). The fate of the unrecovered markers is not clear. Although unlikely, it is possible that the markers were overlooked in the fecal sample screening process or that they were spat out by the animals, but not found on the enclosure floor. It is also possible that the markers adhered to intestinal villi and were not defecated with the other markers. A lack of 100% recovery of markers is common in mammal digestion trials [47], [55].

The mean retention time (MRT) of markers in *A. binturong* was 6.5 h (SD 0.3; range 3.3–9.3 h) and in *P. flavus* 2.5 h (SD 1.6; range 0.7–5.6 h). Defecation patterns of markers were consistent with the short digestive passage times in the two species: markers were defecated in either one (84/142, 59.2%) or two (58/142, 40.8%) fecal samples.

### Fermentation Parameters

As with the preliminary experiment for methane detection (see Table S1), we found no evidence of bacterial fermentation activity in any of the *in vitro* fermentation experiments; culture samples were processed and run on gas chromatography at 0 h, 24 h, and 48 h (depending on experiment), but no peaks were detected for any short chain fatty acid (*acetate, propionate, isobutyrate, butyrate, isovalerate, valerate*) (Table 2).

**Table 2:** Results of *in vitro* experiments, including pH, methane, fiber disappearance, and short chain fatty acid (SCFA) profiles.

*In vitro* variable	*Potos flavus*	*Potos flavus*	*Potos flavus*	*Arctictis binturong*
	March 2009	September 2009	February 2010	February 2010
**pH**				
0 h	6.80 (0.1)	7.87 (0.03)	–	–
24 h	–	3.92 (0.06)	4.50 (0.00)	4.33 (0.06)
48 h	4.48 (0.03)	–	–	–
**pH (blank)**				
0 h	6.63 (0.04)*	9.23 (0.06)	–	–
24 h	–	8.43 (0.21)	6.73 (0.04)*	6.50 (0.00)*
48 h	6.55 (0.07)*	–	–	–
**methane nmol/ml**				
0 h	24.56 (3.5)	23.70 (1.37)	–	–
24 h	–	17.81 (0.42)	26.35 (1.34)	21.28 (2.72)
48 h	24.54 (0.35)	–	–	–
**methane nmol/ml (blank)**				
0 h	20.82 (9.74)	24.81 (3.02)	–	–
24 h	–	26.90 (10.35)	24.20 (3.75)*	20.47 (3.08)*
48 h	25.94 (0.66)	–	–	–
***In vitro*** ** DM disappearance, %**				
0 h	58.32 (1.30)	58.61 (2.90)	–	–
24 h	–	57.59 (6.72)	54.84 (4.93)	36.09 (1.48)
48 h	54.61 (4.97)	–	–	–
**Fiber disappearance, %**				
NDF 0 h	28.16 (6.50)	–	–	–
24 h	–	–	–	–
48 h	14.22 (6.87)	–	–	–
ADF 0 h	25.54 (3.46)	–	–	–
24 h	–	–	–	–
48 h	28.82 (6.03)	–	–	–
**Fecal fiber, %**				
NDF	35.78 (1.57)	–	–	–
ADF	13.87 (0.67)	–	–	–
**Total SCFA (mM)**	*None Detected*	*None Detected*	*None Detected*	*None Detected*
**Individual SCFA (mM)**	*None Detected*	*None Detected*	*None Detected*	*None Detected*
Acetate	*None Detected*	*None Detected*	*None Detected*	*None Detected*
Propionate	*None Detected*	*None Detected*	*None Detected*	*None Detected*
Isobutyrate	*None Detected*	*None Detected*	*None Detected*	*None Detected*
Butyrate	*None Detected*	*None Detected*	*None Detected*	*None Detected*
Isovalerate	*None Detected*	*None Detected*	*None Detected*	*None Detected*
Valerate	*None Detected*	*None Detected*	*None Detected*	*None Detected*

Culture samples were processed and run on gas chromatography at 0, 24, and 48 hours. Standard deviations reported in parentheses. DM  =  Dry Matter; NDF  =  Neutral detergent fiber; ADF  =  Acid detergent fiber; “–”  =  lack of data due to insufficient fecal substrate. * = n = 2 fermentation bottles due to insufficient fecal substrate, all others n = 3.

In the March 2009 *P. flavus* analysis, methane was similar at both 0 and 48 hours (24.56 *versus* 24.54 nmol/ml). In the September 2009 *P. flavus* experiment, methane levels decreased (23.70 *versus* 17.81 nmol/ml). In the February 2010 experiment on *P. flavus* and *A. binturong*, methane levels for both species were similar to the blanks (*P. flavus versus* blank: 26.35, 24.20 nmol/ml; *A. binturong versus* blank: 21.28, 20.47 nmol/m).

In all three experiments, for both species, pH level decreased from the start of the trial to either the 24 h or 48 hour time periods. In the March 2009 *P. flavus* analysis, pH declined from 6.8 at 0 h to 4.48 at 48 h. The September 2009 *P. flavus* experiment revealed a similar pattern: 7.87 at 0 h to 3.92 at 24 h. For the February 2010 experiment, comparisons between the blank and the fecal inoculum are consistent with this acidification: pH for the *P. flavus* blank at 24 h is 6.73 compared to 4.50, while the *A. binturong* blank at 24 h is 6.5 compared to 4.33.

The diet of *A. binturong* comprised 17.5% NDF and 6.7% ADF, and of *P. flavus* 11.9% NDF and 3.1% ADF. *P. flavus* fecal fiber comprised 35.8% NDF and 13.8% ADF. *In vitro* DM disappearance (IVDMD) and fiber disappearance are indicative of the amount of substrate used by microbes during fermentation. Across all experiments, at 0 h the IVDMD values ranged from 54.6–58.6%. Incubation of fecal cultures from either species, irrespective of incubation times (i.e., 24 h or 48 h) resulted in similar DM and fiber disappearance. In estimating dry matter disappearance, the soluble components are not accounted for in the analysis. The absence of DM or fiber disappearance is consistent with the lack of fermentation reported in all experiments.

## Discussion

Important caveats need to be noted regarding methods and research design. Unquestionably, our sample sizes are small; this was unavoidable because of the scarcity of the study species in captivity and difficulties of fecal sample collection. In addition, captive mammals can have decreased intestinal wall area and are less active than wild animals – both differences in gut area and energy expenditure can influence retention times [47], [56]–[58]. In addition, because the animals were born in captivity, intestinal microbial communities no doubt differ from those of their wild counterparts. Indeed, previously we documented a “captivity effect” on *in vitro* carbohydrate fermentation in fecal samples from captive *Gorilla gorilla gorilla* [49]. While inherent limitations with the *in vitro* assay may be a potential issue (variability increases when incubation times are short), we view this as unlikely for two important reasons. First, the fermentation experiments were run on *P. flavus* fecal samples on three separate occasions with all experiments yielding the same result. In addition, previously we employed the same laboratory protocol and equipment on five primate species; this earlier work revealed extensive bacterial fermentation and methane production [49]. In short, it is our perspective that methodological concerns are mitigated by the facts that our research design and sample sizes are consistent with other studies [23], [28], [43], [44], [48], that no data exist on the digestive physiology of the study species (one of which is endangered: *A. binturong*), and that even a small data set can help substantially to clarify the influence of digestion on feeding biology [1], [28], [59], [60].

### Carnivoran solutions to the challenge of frugivory

That we found no evidence of polysaccharide fermentation was unexpected given the study species’ predominantly plant-based diets (both in the wild and captivity) and that other carnivoran species produce short chain fatty acids [29], [61], [62]. The digestive retention times are somewhat more in line with what we expected given their simple, carnivoran gut structure [55], but still shorter than predicted for mammals of their body mass and diet. Indeed, long passage times are certainly not precluded by carnivoran gut anatomy and can be under natural selection pressure in response to a plant-based diet; the omnivorous arctic fox (*Alopex lagopus*; 2.7–4.5 kg), for example, is reported to have passage times of up to 52 h [63].

The digestive passage times documented in the study animals may serve to maintain nitrogen levels by facilitating continuous and high intake of low-N plant foods, although this clearly remains to be tested. Fast passage also certainly influences patterns of carbohydrate extraction [8], [9], and it is noteworthy that the pH dropped appreciably in all experiments between 0, 24, and 48 hours. These results are suggestive of the presence of bacteria that use soluble sugars and less refractory carbohydrates; *Bifidobacteria* spp, for example, use plant-derived fructo-oligosaccharides and thereby produce both lactic and acetic acids that acidify intestinal environments [64]. Both enzymatic digestion and microbial fermentation take time, and even catalytic digesters require sufficient time for enzyme production and nutrient transporter uptake as intestinal rates of hydrolysis and absorption is rate limiting [20]. This explains why other similarly-sized frugivorous mammals have gut passage times that, while rapid compared to hind- or foregut fermenting mammals, are still greater than 10 hours long (e.g., 16 h in *Caluromys philander*) [23]. The mean digestive retention times of *P. flavus* and *A. binturong* were 2.5 and 6.5 hours, respectively – considerably shorter than predicted by the retention times of the similarly-sized, sympatric, nocturnal and highly frugivorous *Caluromys philander* [23], [37].

Such fast digestive passage can be useful for ensuring intake of nutrients in low concentration (e.g., nitrogen), but can leave a mammal of the sizes seen in the study species in a potential ‘energy crisis’ when very ripe fruit is not available in sufficient quantities to maintain high soluble carbohydrate intake. However, these shortfalls may be offset metabolically in the study species [32]. Indeed, while most Carnivora are hypermetabolic, both *P. flavus* and *A. binturong* exhibit hypometabolic adaptations [21], [32], [65], [66]. *Potos flavus* has a lower basal metabolic rate and r_max_ values than would be predicted for its mass, lowers its body temperature while it sleeps, and shivers as it wakes up each evening in order to return its temperature to an active level [32], [35], [65], [66]. *Arctictis binturong* is the largest endotherm with the ability to reduce their peripheral circulation so that the body becomes divided into a warm “core” and a cool “shell” by reducing thermal conductance without permitting core body temperatures to fall [32]. The reduction in metabolism is often so great at low ambient temperatures that it may be below basal rate of thermoneutrality and is one of the greatest reductions (64%) in mammalian basal metabolic rates [21], [32]. Captive *A. binturong* specimens also have large subcutaneous fat deposits throughout their bodies – but especially around the base of their muscular tails (Hartstone-Rose, unpublished data). It is not clear whether this is a result of a captive diet; indeed, no anatomical description of a wild specimen of these rare animals has referred to this detail of anatomy, but we suggest that subcutaneous fat may be an energy storage adaptation that could allow *A. binturong* to adjust their metabolic demands in response to shifts in fruit availability.

In sum, although sample sizes are small and further research is required, we hypothesize that *P. flavus* and *A. binturong* offset limiting N in their fruit diets by maintaining high intake and rapid digestive passage, and offset limiting availability of carbohydrate energy (derived largely from monosaccharides) via metabolic shifts and subcutaneous fat deposits. These digestive and metabolic solutions indicate adaptation to diet independent of carnivoran phylogenic inertia on gut structure.

### Implications for understanding primate frugivory

Among non-volant, arboreal placental mammals, only a few species of Carnivora (e.g., *Potos flavus, Arctictis binturong*, *Arctogalidia trivigata*, *Nandinia binotata*) and primates can have diets that are almost exclusively frugivorous – at least during some seasons. Some primate species stand out in particular – *Pan* spp and *Ateles* spp, for example, are commonly noted for their specialized frugivory and are called ripe fruit specialists [1], [13], [22], [24], [67]. A comparative understanding of digestive physiology can contribute to a more complete picture of how different taxa have adapted to high levels of fruit-consumption and manage the potential challenges of nitrogen (protein) and energy limitations. *Ateles* spp (spider monkeys) in this instance are particularly heuristic because of their sympatry with *Potos*. Kays [22], for example, has documented 100% dietary overlap in the fruits consumed by spider monkeys and *P. flavus* on Barro Colorado Island, Panama; he notes, too, extensive similarity in social organization and foraging behavior.

In absolute terms, *Ateles* spp have among the fastest digestive passage times documented in primates [1], [28], [42], [68], [69]. Data from *Ateles paniscus*, *A. geoffryroi* and *A. belzebuth* indicate digestive passage times (2.5–5.25 h) that are almost identical to those reported here for *P. flavus* and *Arctictis binturong* (2.5–6.5 h). This is contrast to other similarly-sized primate species (e.g., *Cercopithecus* spp) that have digestive passage times ranging from 38.9–48.8 [42], [45]. In absolute terms, the digestive passage times of *P. troglodytes* are longer than those of *Ateles*, *Potos* and *Arctictis* [43], [70]. However, after controlling for body mass differences, *P. troglodytes* exhibits similarly (i.e., relative to body mass) rapid digestive passage times [42], [43]. Clearly, evaluations of gut passage times in mammals are complicated by the many physiological and anatomical variables that influence digestion [42], [47]. However, digestive passage times to body mass ratios can provide a quick and rough means by which to evaluate digestive retention times among very differently-sized taxa [15], [42]. *Ateles* spp (species average body mass: 7.7 kg) and *P. troglodytes* (species average body mass: 45.0 kg), have the lowest ratios of all primates (0.54 and 0.52, respectively) and are similar to *P. flavus* (0.61) and *A. binturong* (0.34), but different from other primate taxa (e.g., *Cercopithecus* spp ratios: 4.3–11.2) [42], [43], [45], [67], [69].

In the case of the two carnivorans, the energetic costs of digesting fruit so quickly may be offset metabolically. Anthropoid primates, however, are not hypometabolic, suggesting that frugivorous taxa such as *Ateles* spp may be particularly efficient at quickly digesting mono- and di-saccharides. Recent research also suggests that *Ateles* spp leverage protein intake over total daily energy intake [13]. In contrast to large-bodied, folivorous *Gorilla beringei* that prioritizes consumption of non-protein energy[14], *Alteles chamack* regulates dietary intake to maintain a consistent daily protein and energy gain [13]. Digestive retention times are consistent with these differences (*G. g. gorilla*: 72 h; *Ateles* spp: 4.2 h) [28], [42], [44], [68], [69].

Do the digestive strategies of the study species converge on strategies exhibited in similarly-sized, omnivorous/frugivorous primate species? We would argue not generally. Overall, digestive strategies among primates exhibit more flexibility in microbial fermentation, anatomy and digestive retention times than in Carnivora ([1], [19], [42]. Thus, while *P. flavus* and *A. binturong* emphasize soluble carbohydrates and do not have the digestive efficiency to take advantage of structural polysaccharides, all primate species studied to date, regardless of gut structure or diet, exhibit high net production of short chain fatty acids from fiber fermentation [29], [49], [70]–[74]. Having the ability to access the energy yielded from soluble carbohydrates *and* structural polysaccharides increases feeding flexibility and total trophic niche space, even in fruit specialists such as *Ateles* and *Pan* [70]. For example, *A. geoffroyi* can consume a diet seasonally predominated by leaves and leaf buds, and the annual diet of highly frugivorous *P. troglodytes* can comprise high levels of terrestrial herbaceous vegetation during some seasons [67], [75], [76]. The comparative data suggest that fermentation and energy yield from short chain fatty acids facilitates dietary breadth for primates - they can handle structural polysaccharides without compromising ability to consume other carbohydrate types.

## Supporting Information

Table S1 **Methane concentration in culture bottles incubated with** ***Potos flavus*** **and** ***Arctictis binturong*** **feces as substrate (December 2008).** This was a preliminary run and methane was the only measurement analyzed to determine presence of fermentation activity. The experiment was terminated based on the absence of any methane production at 24 h.
(DOCX)
