# Supplementary material for: Binturong (Arctictis binturong) and Kinkajou (Potos flavus) Digestive Strategy: Implications for Interpreting Frugivory in Carnivora and Primates
Source: PLoS One. 2014 Aug 26;9(8):e105415. doi: 10.1371/journal.pone.0105415 (PMC4144878; doi:10.1371/journal.pone.0105415)
Supplement: Table S1 — Methane concentration in culture bottles incubated with Potos flavus and Arctictis binturong feces as substrate (December 2008). This was a preliminary run and methane was the only measurement analyzed to determine presence of fermentation activity. The experiment was terminated based on the absence of any methane production at 24 h. (DOCX) [file pone.0105415.s001.docx]

Table S1 Methane concentration in culture bottles incubated with *Potos flavus* and *Arctictis binturong* feces as substrate (December 2008). This was a preliminary run and methane was the only measurement analyzed to determine presence of fermentation activity. The experiment was terminated based on the absence of any methane production at 24 h

| ***In vitro* variable** | ***Potos flavus*** | ***Arctitus binturong*** |
| --- | --- | --- |
| **Treatment^1^** |  |  |
| Methane nmol/ml |  |  |
| 0 hr | 29.53 (3.03) | 36.27 (2.80) |
| 24 | 26.62 (6.04) | 30.42 (4.12) |
|  |  |  |
| **Blank^2^** |  |  |
| Methane nmol/ml |  |  |
| 0 hr | 30.70 (4.41) | 29.78 (1.28) |
| 24 | 26.34 (3.89) | 30.52 (1.26) |
|  |  |  |

^1^ n=6 (3 fermentation bottles/species with substrate, buffer, and fecal inoculum)

^2^ n=4 (2 fermentation bottles/species with substrate and buffer, but no fecal innoculum)
